# Effectiveness of Artificial Intelligence–Assisted Peer Teaching in Orthopedic Clinical Education: Historical Cohort Study

**DOI:** 10.2196/87959

**Published:** 2026-06-24

**Authors:** Chengcheng Yu, Fangcai Li, Ning Zhang, Hejia Hu, Hanxu Huang, Jingkai Wang, Yiqing Tao, Yinan Wu

**Affiliations:** 1 Department of Orthopedics Second Affiliated Hospital of Zhejiang University Hangzhou, Zhejiang China; 2 Zhejiang Cancer Hospital Hangzhou, Zhejiang China

**Keywords:** artificial intelligence, peer teaching, medical education, orthopedic surgery, clinical reasoning, OSCE, Objective Structured Clinical Examination, educational technology, DeepSeek

## Abstract

**Background:**

Peer teaching is an established pedagogical approach in medical education; yet, traditional methods face challenges including inconsistent knowledge support, variable teaching quality, and limited scalability. Artificial intelligence (AI) large language models offer potential to augment peer teaching by providing on-demand access to medical knowledge and clinical reasoning support. However, AI integration within structured peer teaching has not been systematically evaluated in clinical education.

**Objective:**

This study aims to evaluate the effectiveness of AI-assisted peer teaching compared to traditional peer teaching in orthopedic clinical education, with respect to knowledge acquisition, clinical skills development (particularly clinical reasoning), student engagement, and 3-month knowledge retention.

**Methods:**

This historical cohort study compared 2 consecutive cohorts of medical students (aged 20-27 years, 108/190, 56.8% male) at the Second Affiliated Hospital, Zhejiang University School of Medicine, Hangzhou, China. All eligible students from each cohort were enrolled. The control group (2021 cohort, n=96, taught in 2024) received traditional peer teaching; the intervention group (2022 cohort, n=94, taught in 2025) received AI-assisted peer teaching with access to DeepSeek-V3. Primary outcomes were assessed using a validated 50-item multiple-choice examination (0-100 points) and a 4-station Objective Structured Clinical Examination (OSCE; 0-100 points) with standardized rubrics (intraclass correlation coefficient>0.85). Secondary outcomes included student engagement and satisfaction (5-point Likert scales) and AI usage metrics. Assessments were conducted at baseline, postintervention (8 weeks), and 3-month follow-up. Analysis of covariance adjusted for baseline knowledge, prior AI experience, and learning interest to address observed baseline imbalances.

**Results:**

Using independent samples *t* tests (α=.05, 2-tailed), the AI-assisted group demonstrated significantly higher postintervention knowledge scores (mean 79.69, SD 8.41 vs mean 75.33, SD 9.26; mean difference=4.36, 95% CI 1.84-6.87; *P*<.001; Cohen *d*=0.49). OSCE total scores were significantly higher (mean 80.95, SD 7.57 vs mean 76.24, SD 9.23; mean difference=4.71, 95% CI 2.31-7.11; *P*<.001; *d*=0.56), with clinical reasoning showing the largest effect (mean difference=2.22, 95% CI 1.18-3.25; *P*<.001; *d*=0.61). Analysis of covariance adjusted results remained significant for all primary outcomes (adjusted knowledge difference=3.52, *P*=.002; adjusted OSCE difference=4.52, *P*<.001). At 3-month follow-up (174/190, 91.6%), the AI-assisted group maintained higher knowledge scores (mean 77.36, SD 8.60 vs mean 72.84, SD 10.42; mean difference=4.52, 95% CI 1.68-7.36; *P*=.002; *d*=0.47), with similar knowledge decay rates between groups.

**Conclusions:**

This study provides the first systematic evidence that integrating AI tools within structured peer teaching enhances orthopedic clinical education across multiple domains, including knowledge acquisition, OSCE performance, and student engagement. Unlike prior studies examining AI as a stand-alone learning tool, this work demonstrates the synergistic potential of combining AI knowledge support with peer teaching’s social learning benefits, with particularly strong effects on clinical reasoning. These findings support scalable, cost-effective implementation of AI-augmented peer teaching, though randomized controlled trials are needed to confirm causality and determine optimal implementation strategies.

## Introduction

### Background and Rationale

Peer teaching, defined as students learning from and with each other, has emerged as a valuable pedagogical approach in medical education [1,2]. The principles of cognitive and social congruence, which posit that peer teachers may better understand and address learners’ difficulties due to shared recent learning experiences, underlie the effectiveness of peer teaching [3]. Meta-analyses have demonstrated that peer teaching is as effective as faculty teaching for knowledge acquisition while offering additional benefits including enhanced communication skills, professional identity development, and cost-effectiveness [4,5]. These advantages are particularly relevant in resource-constrained clinical education settings, where faculty shortages and increasing student enrollment create growing pressure on traditional teaching capacity [6].

Despite its benefits, traditional peer teaching faces several limitations. Peer teachers may lack comprehensive medical knowledge and clinical experience, potentially propagating misconceptions if not properly supervised [7]. The quality of peer teaching varies considerably depending on individual peer teachers’ preparation and teaching abilities [8]. Peer teachers may struggle to provide evidence-based answers to complex clinical questions on demand, particularly in specialized fields like orthopedic surgery. The scalability of peer teaching is constrained by the availability of trained peer teachers and supervision requirements [9].

These challenges are amplified in orthopedic clinical education, where students must integrate knowledge across multiple domains including anatomy, biomechanics, radiology, and surgical decision-making [10]. Studies have consistently demonstrated that medical students exhibit inadequate musculoskeletal competency upon graduation, with the majority failing basic musculoskeletal knowledge assessments [11]. The cognitive complexity of orthopedic clinical reasoning, requiring simultaneous consideration of mechanism of injury, imaging findings, patient comorbidities, and treatment alternatives, makes it particularly challenging for peer teachers to address learners’ questions comprehensively without access to a robust, on-demand knowledge support system. Orthopedic education therefore represents an ideal context for testing whether AI tools can meaningfully augment peer-mediated learning in a cognitively demanding clinical specialty.

Recent advances in artificial intelligence (AI), particularly large language models (LLMs), offer potential solutions to these challenges. AI tools can provide instant access to medical knowledge, assist with differential diagnosis, explain complex concepts, and support clinical reasoning development [12,13]. Studies have demonstrated that LLMs can perform at the level of medical students or junior physicians on standardized medical examinations [14], and preliminary evidence suggests that AI-assisted learning can improve examination performance and clinical decision-making skills in medical education [15,16]. AI tools have also shown promise in generating clinical reasoning prompts and modeling systematic diagnostic approaches [17,18]. However, concerns regarding AI-generated inaccuracies (“hallucinations”) and the risk of cognitive offloading highlight the importance of structured implementation with appropriate quality safeguards [19]. The integration of AI tools specifically within peer teaching frameworks, where AI’s knowledge capabilities could complement peer teaching’s social learning advantages, remains underexplored. Given that orthopedic surgery requires integration of complex anatomical knowledge, biomechanical principles, and clinical decision-making, it represents an ideal test case for evaluating whether AI can meaningfully augment peer-mediated learning in a cognitively demanding clinical specialty [10].

From a theoretical perspective, the integration of AI within peer teaching can be understood through the lens of distributed cognition theory, which posits that cognitive processes are shared across individuals, tools, and the environment [20]. In an AI-assisted peer teaching model, the AI tool serves as a cognitive artifact that extends the collective knowledge base available during peer interactions, potentially transforming the quality of peer discourse. Complementarily, social constructivist learning theory suggests that knowledge is actively constructed through social interaction [21]; AI may enrich these interactions by providing a shared, accessible knowledge resource that both peer teachers and learners can draw upon to deepen their understanding. This theoretical framework predicts that AI integration would enhance not only individual knowledge acquisition but also the collaborative learning dynamics that distinguish peer teaching from self-directed study.

### Knowledge Gaps

While both peer teaching and AI-assisted learning have been independently studied, their synergistic combination has not been systematically evaluated. Existing literature has not addressed whether AI tools can augment the strengths of peer teaching while mitigating its limitations. Furthermore, most studies on AI in medical education focus on individual learning, neglecting the collaborative and social learning aspects central to peer teaching [22]. There is also limited evidence on the long-term retention of knowledge acquired through AI-assisted educational interventions and on the differential effects of AI across clinical skill domains. This gap is particularly significant because the potential synergy between AI and peer teaching is theoretically compelling: AI could address peer teaching’s knowledge limitations while peer teaching could mitigate AI’s lack of social and motivational dimensions; however, this hypothesis has not been empirically tested. Furthermore, no study has examined whether AI-assisted peer teaching differentially affects higher-order clinical competencies such as clinical reasoning compared to lower-order knowledge recall—a distinction with important implications for curriculum design and AI implementation strategies in medical education.

### Objectives and Hypotheses

This study aimed to evaluate the effectiveness of AI-assisted peer teaching compared to traditional peer teaching in orthopedic clinical education. Specifically, we tested four hypotheses: (Hypothesis 1) AI-assisted peer teaching would enhance knowledge acquisition and performance on the Objective Structured Clinical Examination (OSCE); (Hypothesis 2) the intervention would particularly improve clinical reasoning skills; (Hypothesis 3) AI integration would increase student engagement and satisfaction; and (Hypothesis 4) learning advantages would persist at 3-month follow-up.

## Methods

### Study Design

This historical cohort study compared 2 consecutive cohorts of medical students receiving orthopedic clinical education at Zhejiang University School of Medicine Affiliated Second Hospital. The study followed the STROBE (Strengthening the Reporting of Observational Studies in Epidemiology) guidelines for reporting observational studies [23] and the JARS-Quant (Journal Article Reporting Standards for Quantitative Research) checklist [24].

### Inclusion and Exclusion Criteria

Inclusion criteria were (1) enrollment in the required orthopedic clinical rotation at the Second Affiliated Hospital, Zhejiang University School of Medicine, Hangzhou, China; (2) completion of all required assessments (baseline, postintervention, and OSCE); and (3) provision of written informed consent. Exclusion criteria were (1) previous orthopedic clinical experience beyond the standard curriculum; (2) incomplete assessment data; or (3) withdrawal from the study.

### Participant Characteristics

Participants were medical students from 2 consecutive academic cohorts. The control group comprised 2021 cohort students (n=96) who completed the orthopedic rotation in 2024, and the intervention group comprised 2022 cohort students (n=94) who completed the rotation in 2025. Baseline demographic and educational characteristics, including age, gender, prior AI experience, baseline knowledge scores, and self-reported learning interest, were collected and compared between groups (Table 1). Participants were medical students from 2 consecutive academic cohorts. The control group comprised 2021 cohort students (n=96) who completed the orthopedic rotation in 2024, and the intervention group comprised 2022 cohort students (n=94) who completed the rotation in 2025. Baseline demographic and educational characteristics, including age, gender, prior AI experience, baseline knowledge scores, and self-reported learning interest, were collected and compared between groups (Table 1).

**Table 1:** Baseline characteristics of medical students in traditional peer teaching (control, n=96, 2024 cohort) and artificial intelligence–assisted peer teaching (intervention, n=94, 2025 cohort) groups in orthopedic clinical education, Zhejiang University Affiliated Second Hospital, Hangzhou, China.

Characteristics	Control (n=96)	Intervention (n=94)	Test statistics	*P* value
Age (years), mean (SD)	23.43 (1.75)	23.13 (1.95)	*t*_188_=1.12	.27
Male, n (%)	52 (54.2)	56 (59.6)	*χ*²_1_=0.37	.54
Prior AI^a^ experience, n (%)	36 (37.5)	20 (21.3)	*χ*²_1_=5.26	.02
Baseline knowledge, mean (SD)	66.77 (9.71)	67.92 (9.75)	*t*_188_=−0.82	.42
Learning interest, mean (SD)	3.98 (0.77)	3.71 (0.82)	*t*_188_=2.31	.02

^a^AI: artificial intelligence.

### Sampling Procedures

The study used universal sampling: all eligible students from each consecutive academic cohort enrolled in the required orthopedic clinical rotation were included. Data were collected at the Second Affiliated Hospital, Zhejiang University School of Medicine, Hangzhou, China, during the 2024 academic year (control group) and the 2025 academic year (intervention group). No recruitment incentives or monetary compensation were provided. The study used universal sampling: all eligible students from each consecutive academic cohort enrolled in the required orthopedic clinical rotation were included. Data were collected at the Second Affiliated Hospital, Zhejiang University School of Medicine, Hangzhou, China, during the 2024 academic year (control group) and the 2025 academic year (intervention group). No recruitment incentives or monetary compensation were provided.

### Sample Size, Power, and Precision

Based on previous peer teaching studies showing effect sizes of *d*=0.4-0.6 [25], we calculated that 88 participants per group would provide 80% power to detect a medium effect size (*d*=0.5) at α=0.05 (2-tailed). Our achieved sample sizes (n=96 and n=94) exceeded this requirement, providing adequate statistical power for the primary analyses. Based on previous peer teaching studies showing effect sizes of *d*=0.4-0.6 [25], we calculated that 88 participants per group would provide 80% power to detect a medium effect size (*d*=0.5) at α=0.05 (2-tailed). Our achieved sample sizes (n=96 and n=94) exceeded this requirement, providing adequate statistical power for the primary analyses.

### Educational Interventions: Traditional Peer Teaching (Control Group)

The control group participated in a structured peer teaching program. Students were organized into small groups of 6-8 learners with 2 designated peer teachers (senior students who had completed the orthopedic rotation with distinction). Peer teaching sessions occurred twice weekly for 90 minutes each over 8 weeks (total: 16 sessions, 24 hours). Sessions covered core orthopedic topics including fracture management, joint disorders, spinal conditions, musculoskeletal infections, and orthopedic emergencies, with case discussions, physical examination demonstrations, and diagnostic reasoning exercises. Faculty members provided indirect supervision through presession briefings and postsession feedback, with direct observation in approximately 25% of sessions. Peer teachers had access to standard textbooks, clinical guidelines, and pre-prepared teaching materials developed by faculty.

### AI-Assisted Peer Teaching (Intervention Group)

The intervention group received the same structural framework with integrated AI support. Students and peer teachers had access to DeepSeek-V3 (DeepSeek), a 671-billion-parameter mixture-of-experts LLM, through the official DeepSeek web application (chat.deepseek.com) and mobile app (iOS/Android). Students accessed the platform using individual accounts during the study period from February to June 2025. No custom fine-tuning, institutional configuration, or application programming interface integration was applied; the model was used in its publicly available, standard configuration with default parameters.

AI was integrated into peer teaching in four modes: (1) Presession preparation: peer teachers used AI to review topics, identify common misconceptions, and generate supplementary materials. (2) During sessions, students queried the AI for clarifications, evidence-based information, and alternative explanations. (3) Case-based learning: AI assisted in generating differential diagnoses, explaining pathophysiology, and modeling clinical reasoning. (4) Postsession: students used AI for self-directed study and follow-up questions.

Representative prompts provided during training included: “A 45-year-old male presents with acute onset low back pain radiating to the left leg after lifting. What are the key differential diagnoses and distinguishing features?” (clinical reasoning); “Explain the Garden classification of femoral neck fractures and its clinical significance for treatment decisions” (knowledge clarification); and “In a patient with suspected osteomyelitis, what laboratory and imaging findings support the diagnosis?” (case discussion). Students were encouraged to develop their own queries.

All participants received a 2-hour training session on effective AI use, including prompt formulation, critical evaluation of AI outputs, and recognition of AI limitations. Faculty review of AI interactions was conducted weekly, with approximately 10% of interaction logs randomly sampled and evaluated for factual accuracy by 2 faculty members with orthopedic expertise. Both groups received identical faculty-led lectures, clinical rotations, and patient encounters; peer teaching sessions were supplementary to the core curriculum. Both groups participated in peer teaching sessions of identical duration (16 sessions, 90 minutes each, over 8 weeks; total 24 hours) and frequency; the only difference between groups was the availability of AI support during and outside these sessions. The intervention group additionally received a one-time 2-hour AI training session prior to the start of peer teaching.

### Measures and Covariates

#### Primary Outcomes

Primary outcomes included: (1) Postintervention Knowledge Score, a 50-question validated multiple-choice examination covering orthopedic basic science, clinical diagnosis, treatment principles, and emergency management (0-100 points); (2) Knowledge Gain, calculated as the difference between postintervention and baseline scores; and (3) OSCE Performance, a standardized 4-station OSCE assessing basic science knowledge application (0-25), clinical diagnosis skills (0-35), treatment principles understanding (0-25), and emergency management competencies (0-15), with a total score of 0-100. Additionally, four clinical skills dimensions were evaluated: physical examination, imaging interpretation, history taking, and clinical reasoning (each 0-25). All OSCE stations were evaluated by blinded faculty assessors using standardized rubrics with established interrater reliability (intraclass correlation coefficient>0.85) [26]. OSCE assessors underwent standardized training at the beginning of each assessment period using identical scoring manuals and calibration exercises to ensure consistency across cohorts. The same examination items and OSCE stations (identical case scenarios, scoring rubrics, and standardized patient scripts) were used for both cohorts.

#### Secondary Outcomes

Secondary outcomes included student engagement (discussion participation, question quality, and knowledge sharing) and satisfaction (peer teaching satisfaction, method effectiveness, and overall experience), each measured on 5-point Likert scales; AI usage metrics (weekly frequency, usability, utility, and accuracy perceptions) for the intervention group; and follow-up knowledge retention assessed at 3 months postintervention.

#### Covariates

Covariates included in adjusted analyses were baseline knowledge score (continuous), prior AI experience (dichotomous: yes/no), and self-reported learning interest (continuous, 5-point Likert scale). These were selected based on observed baseline imbalances between groups and their potential to confound the relationship between group assignment and outcomes.

### Analytic Strategy

Based on previous peer teaching studies showing effect sizes of *d*=0.4-0.6 [25], we calculated that 88 participants per group would provide 80% power to detect a medium effect size (*d*=0.5) at α=0.05 (2-tailed). Our achieved sample size (n=96 and n=94) exceeded this requirement.

Normality of continuous outcome variables was assessed using the Shapiro-Wilk test and visual inspection of Q-Q plots. All primary outcomes met normality assumptions (Shapiro-Wilk *P*>.05), except clinical reasoning scores which showed mild nonnormality; Mann-Whitney *U* tests confirmed parametric results. Between-group comparisons used independent samples 2-tailed *t* tests for continuous variables and chi-square tests for categorical variables, with Mann-Whitney *U* tests as sensitivity analyses for Likert-scale data. Effect sizes were calculated using Cohen *d* (interpreted as small [0.2], medium [0.5], or large [0.8]). All tests were 2-tailed with α=.05; 95% CIs are reported for all point estimates [27].

To address baseline imbalances, analysis of covariance (ANCOVA) was performed for all outcomes with baseline knowledge score, prior AI experience (yes/no), and self-reported learning interest as covariates. ANCOVA assumptions (linearity, homogeneity of regression slopes, and normality of residuals) were verified for each model. Missing data patterns were examined; follow-up missingness (8.4%) was assessed for randomness using Little’s missing completely at random (MCAR) test, and sensitivity analyses using multiple imputation (m=20, Rubin’s rules) were conducted. Secondary analyses included Pearson correlations between AI usage and outcomes, and preplanned subgroup analyses by prior AI experience, baseline knowledge tertiles, and gender. All analyses were conducted using Python 3.12 (scipy 1.x, statsmodels 0.14).

### Ethical Considerations

This study was approved by the Institutional Review Board of the Second Affiliated Hospital, Zhejiang University School of Medicine (number Yan2026-0281). Written informed consent was obtained from all participants prior to the study. Students were informed that participation was voluntary, that declining would not affect their academic standing, and that they could withdraw at any time. All data were de-identified using randomly generated participant codes and stored on encrypted institutional servers accessible only to the research team. AI interaction logs were anonymized before analysis. No monetary compensation was provided. No images containing identifiable individuals are included in this manuscript. The educational intervention was implemented as part of an institutionally approved curricular improvement initiative; permission for curricular modifications was obtained from the Office of Medical Education at the Second Affiliated Hospital, Zhejiang University School of Medicine, prior to implementation.

## Results

### Participant Characteristics

**Figure 1:**
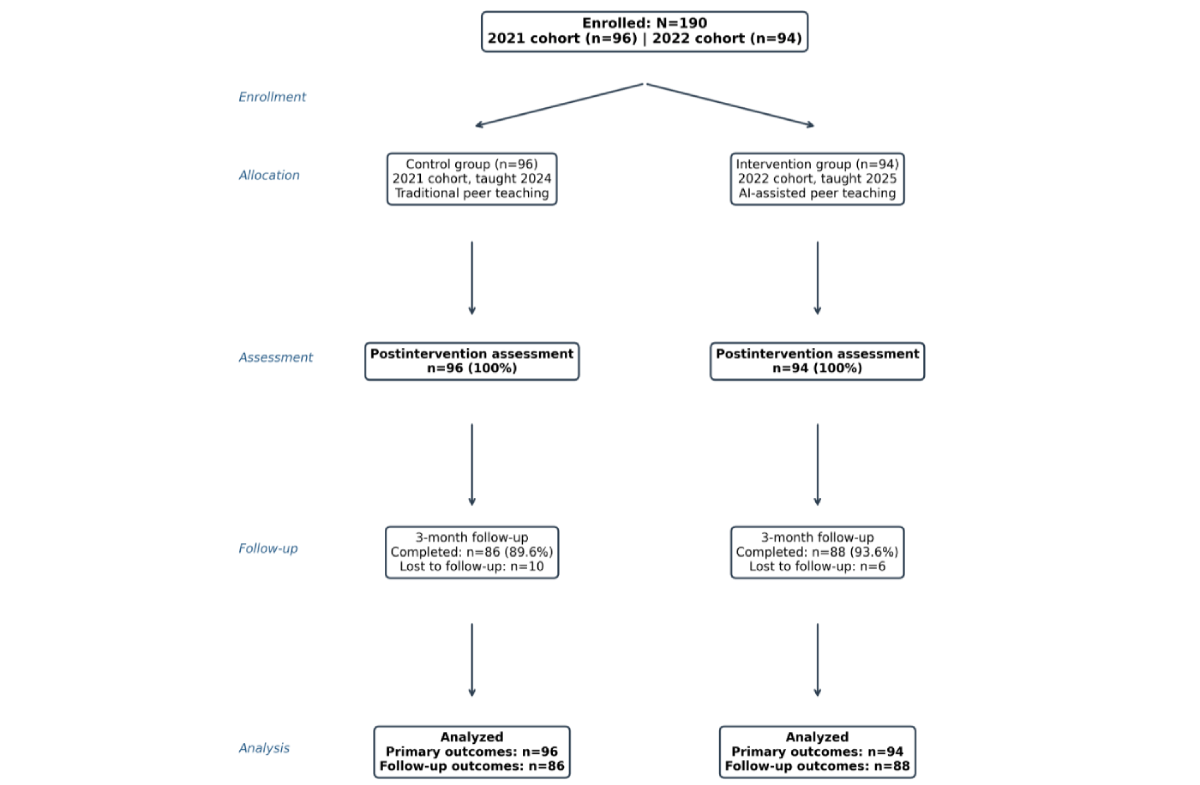
Participant flow diagram for a historical cohort study comparing traditional peer teaching and artificial intelligence-assisted peer teaching among medical students in orthopedic clinical education, Second Affiliated Hospital, Zhejiang University School of Medicine, 2024-2025. All enrolled participants (N=190) completed baseline and post-intervention assessments (100% completion rate). At 3-month follow-up, 174 of 190 participants (91.6%) completed the assessment, with no significant difference in completion rates between groups (χ²1=0.55; *P*=.46). AI: artificial intelligence.

A total of 190 students were enrolled (96 in the control group and 94 in the intervention group). All participants completed baseline and postintervention assessments (100% completion rate). Follow-up data were obtained from 174 participants (91.6% retention rate: control 86/96, intervention 88/94), with no significant difference in follow-up completion between groups (*χ*²_1_=0.55; *P*=.46). The participant flow is depicted in Figure 1.

### Baseline Characteristics

Table 1 presents baseline characteristics. The groups were comparable in age (mean 23.43, SD 1.75 vs mean 23.13, SD 1.95 years; *P*=.27), gender distribution (52/96, 54.2% vs 56/94, 59.6% male; *P*=.54), and baseline knowledge scores (mean 66.77, SD 9.71 vs mean 67.92, SD 9.75; *P*=.42). However, the control group had significantly higher prior AI experience (36/96, 37.5% vs 20/94, 21.3%; *χ*²_1_=5.26; *P*=.02) and baseline learning interest (mean 3.98, SD 0.77 vs mean 3.71, SD 0.82; *P*=.02). These imbalances were addressed through ANCOVA adjustment.

### Primary Outcomes: Knowledge Acquisition

**Figure 2:**
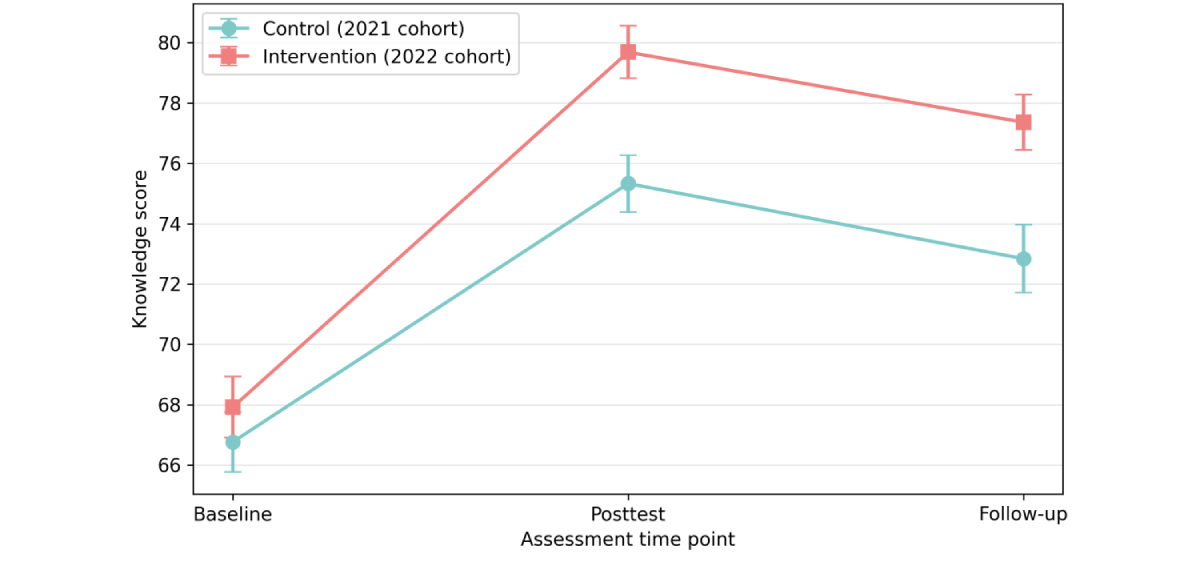
Knowledge development trajectory over time. Mean knowledge scores (SEM) at baseline, postintervention, and 3-month follow-up for control and artificial intelligence–assisted groups. The artificial intelligence–assisted group demonstrated significantly higher scores at both postintervention and follow-up timepoints.

The AI-assisted group demonstrated significantly higher postintervention knowledge scores compared to the control group (mean 79.69, SD 8.41 vs mean 75.33, SD 9.26; mean difference=4.36, 95% CI 1.84-6.87; *t*_188_=3.39; *P*<.001; Cohen *d*=0.49), representing a moderate effect (Figure 2). Knowledge gain from baseline to postintervention was also significantly greater in the AI-assisted group (mean 11.77, SD 8.80 vs mean 8.56, SD 9.19; mean difference=3.20, 95% CI 0.64-5.76; *P*=.02; *d*=0.36).

### OSCE Performance

**Figure 3:**
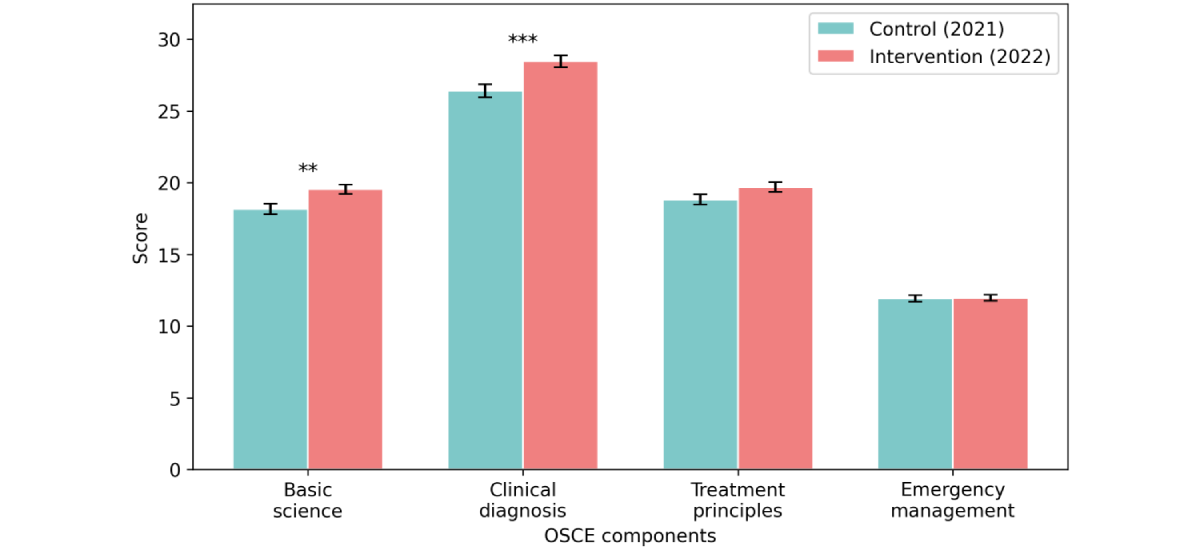
Objective Structured Clinical Examination (OSCE) component scores comparison. Bar chart comparing mean scores (SEM) across OSCE subscales between control and artificial intelligence–assisted groups. Asterisks indicate statistical significance: *P<.05, **P<.01, ***P<.001. Clinical reasoning skills showed the largest improvement in the artificial intelligence–assisted group.

The AI-assisted group achieved significantly higher OSCE total scores (mean 80.95, SD 7.57 vs mean 76.24, SD 9.23; mean difference=4.71, 95% CI 2.31-7.11; t_188_=3.84, *P*<.001; *d*=0.56). Analysis of OSCE component scores revealed differential effects across domains (Table 2, Figure 3). Among knowledge domains, basic science (*P*=.006; *d*=0.41) and clinical diagnosis (*P*<.001; *d*=0.49) showed significant improvements, while treatment principles (*P*=.08) and emergency management (*P*=.89) did not differ significantly. Among clinical skills, clinical reasoning demonstrated the largest effect (t_188_=4.18, *P*<.001; *d*=0.61), followed by physical examination (*P*<.001; *d*=0.54), while imaging interpretation and history taking showed no significant differences.

**Table 2:** Primary and secondary learning outcomes comparing traditional peer teaching (control, n=96) and artificial intelligence–assisted peer teaching (intervention, n=94) in orthopedic clinical education.

Outcome	Control mean (SD)	Intervention mean (SD)	Mean difference	95% CI	*t* test (*df*=188)	*P* value	Cohen *d*
Postintervention knowledge	75.33 (9.26)	79.69 (8.41)	4.36	1.84-6.87	3.39	<.001	0.49
Knowledge gain	8.56 (9.19)	11.77 (8.80)	3.20	0.64-5.76	2.45	.02	0.36
OSCE^a^ total score	76.24 (9.23)	80.95 (7.57)	4.71	2.31-7.11	3.84	<.001	0.56
Basic science	18.17 (3.69)	19.54 (3.07)	1.38	0.41-2.34	2.79	.006	0.41
Clinical diagnosis	26.42 (4.48)	28.47 (3.93)	2.05	0.85-3.25	3.35	<.001	0.49
Treatment principles	18.83 (3.53)	19.71 (3.28)	0.88	−0.09 to 1.85	1.77	.08	0.26
Emergency management	11.93 (2.22)	11.97 (2.04)	0.04	−0.56 to 0.65	0.14	.89	0.02
Physical examination	18.66 (3.59)	20.50 (3.21)	1.84	0.87-2.80	3.72	<.001	0.54
Imaging interpretation	19.34 (2.89)	19.67 (2.98)	0.33	−0.51 to 1.16	0.77	.44	0.11
History taking	20.18 (3.76)	20.51 (3.33)	0.34	−0.67 to 1.35	0.66	.51	0.10
Clinical reasoning	18.06 (4.11)	20.28 (3.12)	2.22	1.18-3.25	4.18	<.001	0.61

^a^OSCE: Objective Structured Clinical Examination.

### Secondary Outcomes: Student Engagement and Satisfaction

**Figure 4:**
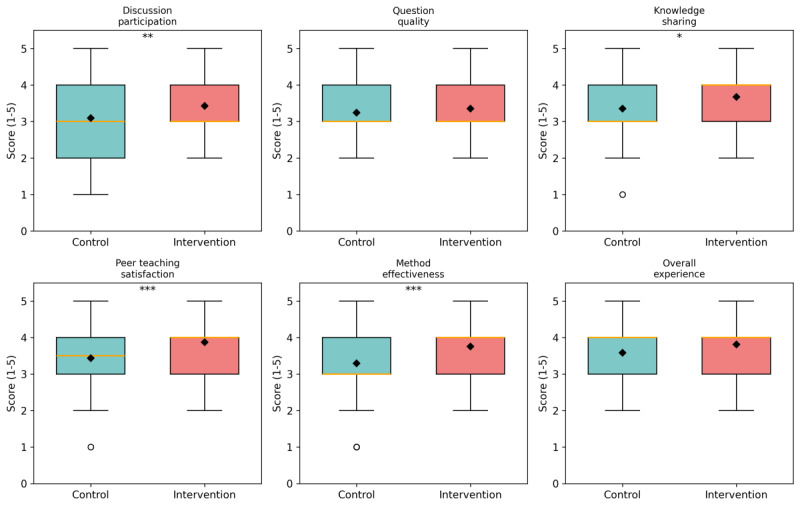
Student engagement and satisfaction outcomes. Box plots showing distribution of engagement and satisfaction measures for both groups. Boxes represent IQR, horizontal lines show medians, and whiskers extend to 1.5×IQR. Individual data points beyond whiskers represent outliers. *P<.05, **P<.01, ***P<.001.

The AI-assisted group demonstrated significantly higher discussion participation (mean 3.43, SD 0.74 vs mean 3.09, SD 0.85; *P*=.005; *d*=0.42) and knowledge sharing (mean 3.67, SD 0.75 vs mean 3.35, SD 0.92; *P*=.01; *d*=0.38). Question quality did not differ significantly (*P*=.31). Peer teaching satisfaction (mean 3.87, SD 0.69 vs mean 3.44, SD 0.86; *P*<.001; *d*=0.56) and perceived method effectiveness (mean 3.76, SD 0.68 vs mean 3.29, SD 0.92; *P*<.001; *d*=0.57) both showed moderate effect sizes. Overall experience showed a trend toward improvement (*P*=.08; *d*=0.26). Mann-Whitney *U* sensitivity analyses for all Likert-scale measures produced results consistent with parametric tests (Table 3 and Figure 4).

**Table 3:** Student engagement and satisfaction outcomes.

Measures^a^	Control mean (SD)	Intervention mean (SD)	95% CI	*t* test (*df*=188)	*P* value	Cohen *d*
Discussion participation	3.09 (0.85)	3.43 (0.74)	0.11-0.56	2.87	.005	0.42
Question quality	3.24 (0.78)	3.35 (0.74)	−0.10 to 0.33	1.01	.31	0.15
Knowledge sharing	3.35 (0.92)	3.67 (0.75)	0.08-0.55	2.59	.01	0.38
Peer teaching satisfaction	3.44 (0.86)	3.87 (0.69)	0.21-0.66	3.85	<.001	0.56
Method effectiveness	3.29 (0.92)	3.76 (0.68)	0.23-0.69	3.95	<.001	0.57
Overall experience	3.58 (0.90)	3.81 (0.83)	−0.02 to 0.47	1.79	.08	0.26

^a^All measures rated on 5-point Likert scale (1=strongly disagree, 5=strongly agree).

### AI Usage Patterns and Quality Perceptions

**Figure 5:**
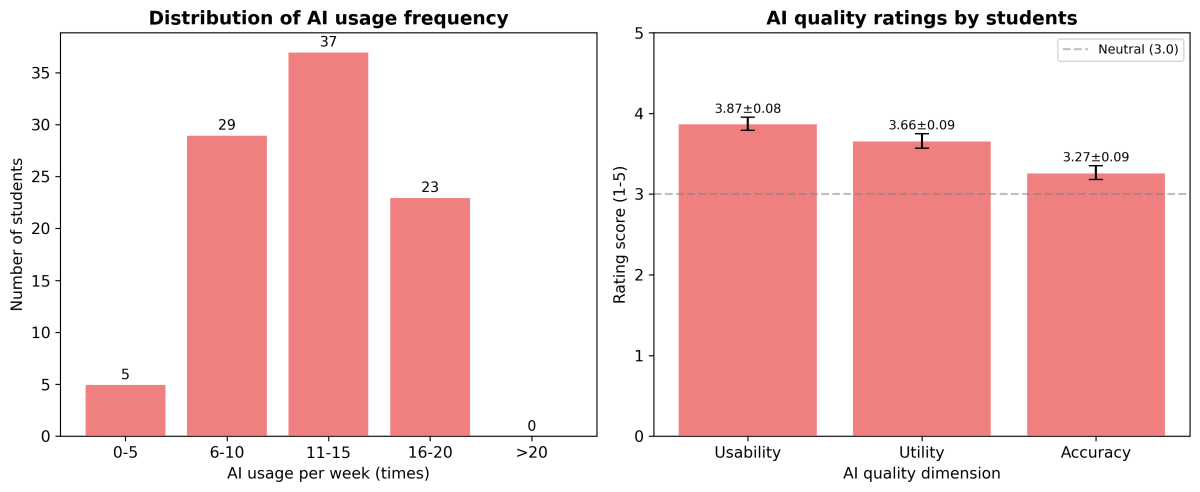
Artificial intelligence usage patterns and quality ratings. The left panel shows the frequency distribution of weekly artificial intelligence usage among intervention group students. The right panel displays mean ratings (SEM) for three quality dimensions: usability, utility, and accuracy, all measured as subjective ratings on 5-point scales. AI: artificial intelligence.

In the intervention group, students used the AI tool an average of 12.30 (SD 4.01) times per week (median 12, IQR 5-20). Most students (39.4%) used AI 11-15 times weekly; no students exceeded 20 uses, suggesting judicious rather than excessive use. Usability received the highest subjective quality rating (mean 3.87, SD 0.79), followed by utility (mean 3.66, SD 0.87) and perceived accuracy (mean 3.27, SD 0.83). It should be noted that these quality ratings reflect students’ subjective perceptions rather than objective measures of AI output quality. AI utility perception was positively associated with postintervention knowledge (*r*=0.29; *P*=.004), while usage frequency showed no significant correlation (*r*=−0.08; *P*=.44), suggesting that engagement quality rather than quantity drove learning benefits (Figure 5).

### Adjusted Analyses

After controlling for baseline knowledge, prior AI experience, and learning interest using ANCOVA, all primary findings remained statistically significant (Table S1 in Multimedia Appendix 1). Adjusted postintervention knowledge scores remained significantly higher in the AI-assisted group (adjusted mean difference=3.52, 95% CI 1.30-5.74; *P*=.002). Adjusted OSCE total scores showed a similar pattern (adjusted mean difference=4.52, 95% CI 2.07-6.97; *P*<.001). The adjusted effect for clinical reasoning remained the largest (adjusted mean difference=2.10, 95% CI 1.04-3.15; *P*<.001). Baseline knowledge was a significant covariate across all knowledge-related models (*P*<.001), while prior AI experience (*P*=.56 for knowledge; *P*=.73 for OSCE) and learning interest (*P*=.38 for knowledge; *P*=.86 for OSCE) were not significant covariates for any outcome, indicating that the observed baseline imbalances did not confound group differences.

### Missing Data and Follow-up Knowledge Retention

Follow-up noncompleters (n=16, 8.4%) did not differ significantly from completers on baseline knowledge (*P*=.53), postintervention knowledge (*P*=.38), learning interest (*P*=.64), or group allocation (*χ*²_1_=0.55, *P*=.46), consistent with the MCAR pattern. Little’s MCAR test formally confirmed this assumption (*χ*²_7_=7.67; *P*=.36). At 3-month follow-up, the AI-assisted group maintained significantly higher knowledge scores (mean 77.36, SD 8.60 vs mean 72.84, SD 10.42; mean difference=4.52, 95% CI 1.68-7.36; *P*=.002; *d*=0.47). Knowledge decay from postintervention to follow-up was similar between groups (intervention: 2.43 points; control: 2.64 points; *P*=.85). Multiple imputation sensitivity analyses (m=20, Rubin’s rules) produced consistent results (adjusted difference=3.71, 95% CI 1.00-6.41; *P*=.008).

### Subgroup Analyses

Subgroup analysis by prior AI experience revealed no significant interaction effect (*P* for interaction=.42). When stratified by baseline knowledge tertiles, effects were largest in the low-baseline subgroup (*d*=0.65), followed by middle (*d*=0.54) and high (*d*=0.37) tertiles. No significant gender differences were observed (*P* for interaction=.68).

## Discussion

### Principal Findings

This historical cohort study evaluated the effectiveness of AI-assisted peer teaching in orthopedic clinical education against four prespecified hypotheses. The findings supported all four hypotheses: students in the AI-assisted group demonstrated superior knowledge acquisition and OSCE performance (Hypothesis 1), with particularly pronounced improvement in clinical reasoning skills (Hypothesis 2); the AI-assisted group showed greater engagement and satisfaction (Hypothesis 3); and the learning advantages persisted at 3-month follow-up (Hypothesis 4). After adjusting for baseline imbalances using ANCOVA, all primary findings remained statistically significant, confirming the robustness of the observed effects [25].

These findings are noteworthy in the context of orthopedic education, where students frequently struggle with the integration of basic science and clinical knowledge required for musculoskeletal assessment and management [10,11]. The moderate to large effect sizes observed across multiple outcome domains suggest that AI augmentation addresses a genuine instructional gap in peer teaching rather than producing improvements attributable solely to novelty or increased engagement.

### Interpretation of Findings: Knowledge Acquisition and Clinical Reasoning

The improvement in postintervention knowledge scores represents an educationally meaningful moderate effect, aligning with emerging evidence that AI tools can enhance medical knowledge acquisition in educational settings [14,15]. The superior knowledge gain in the AI-assisted group suggests that AI augmented the learning process by providing immediate access to evidence-based information when questions arose—a capability that peer teachers alone often lack [28]. Notably, the proportion of students achieving excellent scores was higher in the AI-assisted group for both knowledge and OSCE assessments, suggesting that AI shifted the entire performance distribution upward rather than merely benefiting a subset of learners. An important consideration is the direction of potential bias: the control group had higher prior AI experience and learning interest, which would favor the control group. Neither was a significant ANCOVA covariate, and the consistency between unadjusted and adjusted effect sizes further supports the conclusion that baseline differences did not confound the observed effects.

The improvement in clinical reasoning, which showed the largest effect size among all outcomes, warrants particular attention. Clinical reasoning, the cognitive process underlying diagnostic and therapeutic decision-making, is notoriously difficult to teach and assess [29,30]. AI tools may facilitate reasoning development through step-by-step differential diagnosis generation and systematic exploration of diagnostic pathways [17,18]. The differential OSCE improvement pattern is informative: strong effects were observed for clinical reasoning and physical examination, while imaging interpretation and history taking showed no significant differences. This pattern suggests that AI specifically enhances deliberative cognitive processes, tasks that benefit from structured analytical frameworks, rather than skills developed primarily through repeated patient contact or interpersonal communication [10].

These findings have important implications for the strategic deployment of AI in clinical education. The relative improvement in clinical reasoning is particularly notable given this competency’s documented resistance to educational interventions [29]. The ANCOVA-adjusted OSCE effect was consistent with the unadjusted estimate, confirming that baseline imbalances favoring the control group produced conservative unadjusted estimates. AI integration should therefore be targeted toward cognitive tasks where structured knowledge support can scaffold learning, such as diagnostic reasoning, treatment planning, and evidence-based decision-making, rather than applied uniformly across all clinical competency domains.

### Student Engagement and AI Usage Patterns

The AI-assisted group demonstrated higher discussion participation, knowledge sharing, satisfaction, and perceived effectiveness. Rather than displacing peer interaction, a concern frequently raised in the educational technology literature [19], AI appears to have enriched it by providing a richer substrate of information for productive peer exchanges. The nonsignificant difference in question quality suggests that AI enhanced discussion depth without fundamentally altering inquiry patterns. This finding is consistent with the collaborative learning literature suggesting that access to richer information sources can elevate the quality of peer discourse without replacing the social dynamics that make peer teaching effective [31].

An apparent paradox is that students’ subjective perception of AI accuracy was only moderate, yet significant learning improvements were observed. This moderate accuracy rating may reflect well-calibrated critical evaluation skills fostered by the AI training session, which emphasized recognizing AI limitations [19]. The process of identifying and correcting AI errors may itself be pedagogically valuable, as error detection requires and reinforces domain knowledge [32]. This interpretation suggests that the combination of accessible but imperfect AI knowledge with human critical evaluation may be more educationally powerful than either element alone—a finding with implications for how AI tools should be framed in educational contexts.

The relationship between AI usage patterns and learning outcomes merits further consideration. AI utility perception, but not usage frequency, predicted learning outcomes, suggesting that students who engaged more thoughtfully with AI, critically evaluating its outputs, integrating AI-provided information with existing knowledge, and using AI as a springboard for deeper inquiry, derived greater educational benefit. This aligns with the broader educational technology literature indicating that the quality of technology-mediated learning interactions, rather than mere exposure duration, determines educational effectiveness [15,22]. Practical implications include the importance of training students not merely in how to use AI tools, but in how to engage with them critically and reflectively.

### Subgroup Effects and Knowledge Retention

Stratified by baseline knowledge tertiles, the largest effect was observed in the low-baseline subgroup, followed by the middle and high subgroups, consistent with Vygotsky’s zone of proximal development framework: AI may scaffold most effectively for students with the greatest growth potential, while high-performers face ceiling effects on standardized assessments [31]. The absence of significant interaction effects for prior AI experience or gender supports the feasibility of universal implementation, suggesting that AI-augmented peer teaching need not be restricted to technologically experienced students and can benefit learners across diverse demographic subgroups.

At 3-month follow-up, the AI-assisted group maintained higher knowledge scores with similar knowledge decay rates between groups, indicating that AI-assisted learning enhanced initial acquisition rather than fundamentally altering retention mechanisms [33]. The comparable decay rates suggest that AI-assisted learning does not create a dependency that leads to accelerated forgetting once AI access is removed. Rather, the enhanced initial learning appears to be genuinely encoded, consistent with the hypothesis that AI facilitated deeper processing during the learning phase rather than superficial information retrieval.

Whether the clinical reasoning advantages demonstrated under standardized assessment conditions translate into improved diagnostic accuracy and patient outcomes in authentic clinical settings remains an important question requiring longitudinal studies tracking learners into clinical practice [29]. Future studies should also investigate whether spaced retrieval practice combined with AI support could further enhance long-term retention and whether different AI implementation models produce differential learning effects across clinical training stages.

### Limitations

This study has several limitations. First, the historical cohort design precludes definitive causal inference. Despite identical assessment instruments, curriculum content, and faculty assignments, temporal confounders, including evolving institutional emphasis on AI literacy, curriculum drift, and societal AI awareness, cannot be fully excluded. Causal conclusions cannot be definitively drawn; these findings should be interpreted as suggestive evidence requiring confirmation through randomized controlled trials.

Second, this single-center, single-specialty study limits generalizability. Third, subtle differences in assessment conditions across years cannot be entirely ruled out, and assessor blinding could have been compromised if assessors were aware of the AI initiative. Fourth, self-reported engagement and satisfaction measures are susceptible to social desirability and novelty bias. Fifth, total study time, including AI-assisted self-directed learning outside structured peer teaching sessions, was not quantified; therefore, we cannot rule out that the intervention group’s additional unstructured AI usage contributed learning time beyond the equivalent scheduled peer teaching hours. Structured sessions may represent unaccounted additional learning time. Sixth, our quantitative usage log analysis did not capture the sophistication of individual student-AI interactions; qualitative research is needed. Seventh, potential risks of AI-assisted learning, including cognitive offloading and overreliance, were not directly assessed; future studies should include AI-free assessment conditions.

### Conclusions

This study provides the first systematic evidence that integrating AI tools within structured peer teaching can meaningfully enhance orthopedic clinical education. Unlike prior studies examining AI as a stand-alone learning tool, unlike prior studies examining AI as a stand-alone individual learning tool, this work demonstrates the synergistic potential of combining AI knowledge support with peer teaching’s social learning benefits, with particularly strong effects on clinical reasoning—a competency that has historically proven resistant to educational interventions [29,30].

These findings carry practical implications. The intervention required minimal infrastructure (integration of a freely accessible AI tool) and modest training investment (a 2-hour session), suggesting feasibility for widespread adoption. However, implementation challenges include digital equity concerns, the risk of AI overreliance undermining peer interaction, the need for ongoing AI content validation, and faculty development requirements. Institutions should pilot implementations with robust evaluation frameworks before scaling [13,19].

Future research should prioritize randomized controlled trials, multicenter studies across specialties, longitudinal tracking into clinical practice, and qualitative studies of student-AI interaction patterns. As AI tools continue to evolve, the educational community must develop frameworks that maximize learning benefits while preserving the critical thinking and clinical judgment at the core of medical practice [22].

## Appendix

Multimedia Appendix 1 Analysis of Covariance (ANCOVA) Results for All Primary and Secondary Outcomes, Adjusting for Baseline Knowledge, Prior AI Experience, and Learning Interest. Historical Cohort Study Comparing Traditional Peer Teaching (Control, n=96) and AI-Assisted Peer Teaching (Intervention, n=94), Second Affiliated Hospital, Zhejiang University School of Medicine, 2024–2025.

## Abbreviations

AI: artificial intelligence
ANCOVA: analysis of covariance
JARS-Quant: Journal Article Reporting Standards for Quantitative Research
LLM: large language model
MCAR: missing completely at random
OSCE: Objective Structured Clinical Examination
STROBE: Strengthening the Reporting of Observational Studies in Epidemiology

## Data Availability

The data that support the findings of this study are available on reasonable request from the corresponding author. The data are not publicly available due to participant privacy considerations.
