# Supplementary material for: Effectiveness of Artificial Intelligence–Assisted Peer Teaching in Orthopedic Clinical Education: Historical Cohort Study
Source: JMIR Med Educ. 2026 Jun 24;12:e87959. doi: 10.2196/87959 (PMC13347076; doi:10.2196/87959)
Supplement: Multimedia Appendix 1 [file mededu_v12i1e87959_app1.docx]

**Table S1.** Analysis of Covariance (ANCOVA) Results for All Primary and Secondary Outcomes, Adjusting for Baseline Knowledge, Prior AI Experience, and Learning Interest. Historical Cohort Study Comparing Traditional Peer Teaching (Control, n=96) and AI-Assisted Peer Teaching (Intervention, n=94), Second Affiliated Hospital, Zhejiang University School of Medicine, 2024–2025.

| **Outcome** | **Adjusted Difference** | **95% CI** | **P** | **Cohen d** | **Baseline Knowledge P** | **Prior AI Experience P** | **Learning Interest P** |
| --- | --- | --- | --- | --- | --- | --- | --- |
| ***Primary outcomes*** | | | | | | | |
| Post-intervention knowledge | 3.52 | [1.30, 5.74] | .002 | 0.47 | <.001 | .558 | .383 |
| Knowledge gain | 3.52 | [1.30, 5.74] | .002 | 0.47 | <.001 | .558 | .383 |
| OSCE total score | 4.52 | [2.07, 6.97] | <.001 | 0.55 | .001 | .726 | .865 |
| Basic science | 1.15 | [0.21, 2.09] | .017 | 0.36 | <.001 | .530 | .750 |
| Clinical diagnosis | 1.79 | [0.66, 2.92] | .002 | 0.47 | <.001 | .547 | .264 |
| Treatment principles | 0.69 | [−0.27, 1.64] | .161 | 0.21 | <.001 | .131 | .450 |
| Emergency management | −0.12 | [−0.72, 0.49] | .703 | 0.06 | <.001 | .909 | .063 |
| Physical examination | 1.67 | [0.67, 2.67] | .001 | 0.49 | .143 | .948 | .119 |
| Imaging interpretation | 0.35 | [−0.52, 1.22] | .426 | 0.12 | .200 | .179 | .497 |
| History taking | 0.41 | [−0.63, 1.46] | .437 | 0.12 | .049 | .431 | .472 |
| Clinical reasoning | 2.10 | [1.04, 3.15] | <.001 | 0.59 | .002 | .268 | .361 |
| ***Secondary outcomes*** | | | | | | | |
| Discussion participation | 0.45 | [0.23, 0.67] | <.001 | 0.60 | .058 | .202 | <.001 |
| Question quality | 0.17 | [−0.04, 0.39] | .118 | 0.24 | .266 | .202 | <.001 |
| Knowledge sharing | 0.42 | [0.19, 0.66] | <.001 | 0.54 | .652 | .820 | <.001 |
| Peer teaching satisfaction | 0.43 | [0.20, 0.66] | <.001 | 0.56 | .080 | .980 | .585 |
| Method effectiveness | 0.49 | [0.25, 0.73] | <.001 | 0.60 | .228 | .263 | .681 |
| Overall experience | 0.26 | [−0.00, 0.51] | .051 | 0.29 | .250 | .541 | .229 |
| ***Follow-up outcome*** | | | | | | | |
| Follow-up knowledge (n=174) | 3.87 | [1.10, 6.65] | .006 | 0.43 | <.001 | .437 | .382 |

**Note:** Adjusted difference represents the estimated mean difference (intervention minus control) from ANCOVA models. Cohen *d* was calculated as the adjusted difference divided by the square root of the mean squared error. *P* values for covariates indicate their individual contribution to the model. All models included three covariates: baseline knowledge score (continuous), prior AI experience (dichotomous), and self-reported learning interest (continuous). Follow-up analysis was conducted on complete cases (n=174).
